# Clinical relevance of PD-1 positive CD8 T-cells in gastric cancer

**DOI:** 10.1007/s10120-023-01364-7

**Published:** 2023-02-12

**Authors:** Joan Choo, Ley Fang Kua, Mu Yar Soe, Bernadette Reyna Asuncion, Benjamin Kye Jyn Tan, Chong Boon Teo, Ryan Yong Kiat Tay, Jimmy So, Asim Shabbir, Kim Guowei, Hon Lyn Tan, Gloria Chan, Haoran Ma, Gokula Krishnan Ramachandran, Jeffrey H. Y. Lum, Cheng Ean Chee, Sriram Sridharan, Patrick Tan, Raghav Sundar, Wei Peng Yong

**Affiliations:** 1 Department of Haematology-Oncology, National University Cancer Institute, Singapore (NCIS), National University Health System, 1E Lower Kent Ridge Road, Singapore, 119228 Singapore; 2 Cancer Science Institute, National University of Singapore, Singapore, Singapore; 3 Yong Loo Lin School of Medicine, National University of Singapore, Singapore, Singapore; 4 Department of Surgery, National University Hospital, National University Health System, Singapore, Singapore; 5 Cancer and Stem Cell Biology Program, Duke-NUS Medical School, Singapore, Singapore; 6 Singapore Gastric Cancer Consortium, Singapore, Singapore; 7 Department of Pathology, National University Health System, Singapore, Singapore; 8 Department of Physiology, National University of Singapore, Singapore, Singapore; 9 Agency for Science, Technology and Research, Genome Institute of Singapore, Singapore, Singapore; 10 SingHealth/Duke-NUS Institute of Precision Medicine, National Heart Centre Singapore, Singapore, Singapore; 11 The N.1 Institute for Health, National University of Singapore, Singapore, Singapore

**Keywords:** Gastric cancer, CD8 T-cells, PD-1, Multiplex immunohistochemistry

## Abstract

**Background:**

We evaluated the relevance of PD-1^+^CD8^+^ T-cells in gastric cancer (GC) including prognostic significance, association with chemotherapy and immunotherapy sensitivity and correlations with the tumor microenvironment (TME).

**Methods:**

Discovery cohort: GC samples were evaluated for AE1/3, CD8, PD-1, Ki-67 and Granzyme-B expression with fluorescence-based multiplex immunohistochemistry (mIHC). Validation cohorts: we analyzed bulk RNAseq GC datasets from TCGA, the “3G” chemotherapy trial and an immunotherapy phase 2 trial. The cox proportional hazards model was used to identify factors that influenced overall survival (OS). To study the TME, we analyzed single-cell RNAseq performed on GCs.

**Results:**

In the discovery cohort of 350 GCs, increased PD-1 expression of CD8 T-cells was prognostic for OS (HR 0.822, *p* = 0.042). PD-1 expression in CD8 T-cells highly correlated with cytolytic [Granzyme-B^+^] (*r* = 0.714, *p* < 0.001) and proliferative [Ki-67^+^] (*r* = 0.798, *p* < 0.001) activity. Analysis of bulk RNAseq datasets showed tumors with high *PD-1* and *CD8A* expression levels had improved OS when treated with immunotherapy (HR 0.117, *p* = 0.036) and chemotherapy (HR 0.475, *p* = 0.017). Analysis of an scRNAseq dataset of 152,423 cells from 40 GCs revealed that T-cell and NK-cell proportions were higher (24% vs 18% and 19% vs 15%, *p* < 0.0001), while macrophage proportions were lower (7% vs 11%, *p* < 0.0001) in CD8PD-1_high_ compared to CD8PD-1_low_ tumors.

**Conclusion:**

This is one of the largest GC cohorts of mIHC combined with analysis of multiple datasets providing orthogonal validation of the clinical relevance of PD-1^+^CD8^+^ T-cells being associated with improved OS. CD8PD-1_high_ tumors have distinct features of an immunologically active, T-cell inflamed TME.

**Supplementary Information:**

The online version contains supplementary material available at 10.1007/s10120-023-01364-7.

## Introduction

Gastric cancer is the fifth most common cancer with one million new cases annually and more than 750,000 deaths worldwide [1]. Despite combination treatment approaches with surgery and chemo/radiotherapy, prognosis remains poor with an average 5-year survival of 32%. Immune checkpoint inhibitors (ICI) have increasingly been incorporated into the treatment landscape of gastric cancer with significant efficacy observed in the metastatic setting [2–4]. PD-L1 expression may predict for response to ICI, but it is an imperfect biomarker [5, 6]. There is no consensus criteria for assessing PD-L1 status – multiple antibodies assays [2–4, 7, 8] and different scoring systems [2–4, 7] are used. Moreover, the prognostic significance of PD-L1 expression in gastric cancer is controversial with multiple positive and negative studies [9, 10].

Immune cells are known to be a determining factor in tumor initiation, progression, and metastasis; three component phases that have been described are elimination, equilibrium and escape [11]. However, understanding precise mechanisms behind which particular cell types promote or prevent disease in each tumor type is challenging. Studies in epithelial cancer types have looked at the role and activation status of infiltrating CD8 positive T-cells including PD-1 expression in these immune cells. Increased expression of PD-1 in CD8 T cells conferred improved survival outcomes in a variety of epithelial cancer types such as breast, pancreatic, and head and neck tumors [12–14]. The characteristics of CD8 T-cell subsets in the gastric cancer tumor microenvironment and their clinical relevance have not been studied in great depth.

Multiplex immunohistochemistry/immunofluorescence (mIHC/IF) and digital slide image analysis has the benefit of assessing the expression of up to eight proteins using just a single tissue section [15, 16] and has shown concordance to traditional IHC methods [17]. This approach objectively quantifies protein expression within the tumor as well as immune cells, aiming to minimize expression variability and interobserver variability in the evaluation of samples. Furthermore, of particular interest to this study, it can specifically identify populations of double-positive cells.

In this study, we used multiplex immunohistochemistry/immunofluorescence (mIHC/IF) and digital slide image analysis to examine the CD8-positive tumor infiltrating lymphocyte status in gastric cancer samples. In particular, we focus on the correlation between CD8, PD-1 double positive cells, and clinical outcomes such as overall survival. We expand our analysis to study the relevance of these findings in chemotherapy and immunotherapy treated GC trial cohorts, as well as the distinct tumor microenvironments of high CD8PD-1 tumors using single-cell RNAseq.

## Methods

### Discovery cohort

#### Clinical samples

We collected tumor samples from chemotherapy naïve patients who underwent gastrectomy for gastric cancer (GC) in National University Hospital of Singapore between 2000 and 2013 for immunohistochemistry analysis. Post operatively, patients were offered adjuvant treatment if indicated and underwent surveillance. Staging of GC was performed according to the latest American Joint Committee on Cancer (8th edition). This study was approved by the Domain Specific Review Board of the National Healthcare Group of Singapore (Reference 2015/00209).

#### Multiplex immunohistochemistry (IHC) staining of tissue microarray (TMA)

TMA blocks were created from tissue samples where at least 50% of the sample area was tumor based on formal pathological analysis. For each sample, two or three representative tumor cores of 1 mm diameter were transferred from donor FFPE tissue blocks to recipient TMA blocks using the TMA Grand-Master (3DHISTECH Ltd., Budapest, Hungary). Each TMA block contained up to approximately 80 cores (range 40–77). TMA sections were subsequently labelled with antibodies as below.

Multiplex IHC staining was performed using Leica Bond RX machine (Leica Biosystems, Wetzlar, Germany) with Opal Multiplex fIHC kit (Perkin Elmer, Waltham, MA, USA). Three micrometer thick tissue microarray sections were stained with primary antibodies against the following: Cytokeratin (Abcam, clone AE1/AE3, dilution 1:100), CD8 (Novocastra, clone 4B11, dilution 1:3000; Leica Biosystems, Buffalo Grove, IL), Ki67 (Novocastra, PA0118, dilution 1:100; Leica Biosystems, Buffalo Grove, IL), Granzyme B (Novocastra, NCL-L-GRAN-B, dilution 1:100; Leica Biosystems, Buffalo Grove, IL), and PD-1 (clone NAT105,, dilution 1:1000; abcam). Followed by secondary antibodies, fluorophore (FITC, Cy3, Cy5, Texas Red)-conjugated tyramides signal amplification (TSA) buffer (Perkin Elmer) were added. After six sequential reactions, sections were counterstained with DAPI and mounted with Vectashield fluorescence mounting medium (Vector Labs, Burlingame, CA). Human tonsil FFPE tissues stained with or without primary antibody were used as positive and negative controls in each IHC staining.

IHC stains were examined by a pathologist using the Vectra digital slide imaging system and the Inform software (Perkin Elmer, Waltham, MA, USA). Individual cells were graded for CD8, PD-1, Granzyme B, Ki-67 and Cytokeratin) staining on membranous compartments.

For quantitative analysis of CD8 T-cell infiltration, PD-1 positive CD8 T-cells, granzyme-B positive CD8 T-cells and Ki-67 positive CD8 T-cells, we obtained the absolute number of CD8^+^ T-cells, double-positive PD-1^+^CD8^+^, double-positive granzyme-B^+^CD8^+^ and double-positive Ki-67^+^CD8^+^ T-cells present in each TMA sample respectively, divided by total number of tumor cells present within the TMA sample to obtain a normalized ratio for each value.

##### Batch effect analysis

Analysis of the various TMA blocks using principal components analysis of the tumor cell count, CD8 T-cells, granzyme-B positive CD8 T-cells, Ki-67 positive CD8 T-cells, PD-1 positive CD8 T-cells revealed no batch effects (Supplementary Fig. 1).

#### Mismatch repair (MMR) IHC staining

MMR status was evaluated independently, on full stained slides using Ready-to-Use monoclonal antibodies against MLH1 (clone M1, Ventana/Roche, Basal, Schweiz), MSH6 (clone SP93, Ventana/Roche, Basal, Schweiz), MSH2 (clone G219-1129, Ventana/Roche, Basal, Schweiz) and PMS2 (clone A16-4, Ventana/Roche, Basal, Schweiz).

### Validation cohorts

#### TCGA gastric cancer dataset

Transcriptomic gene expression Level 3 RSEM-normalized RNASeqV2 data and clinical data from the TCGA Study of Gastric Adenocarcinoma were extracted from the Broad GDAC Firebrowse [18]. Illumina HiSeq RSEM-normalized RNAseqV2 gene values were used for correlations of *CD8A* and *PDCD1*. The TCGA Study of Gastric Adenocarcinoma cohort was aligned using STAR v2.6.1. Disease free survival (DFS) and overall survival (OS) including censorship data were extracted from the Pan-Cancer immune landscape analysis by Thorsson and colleagues [19].

#### Immunotherapy treated GC trial dataset

This analysis is a sub-study of a larger dataset of bulk RNAseq of gastric cancer previously reported [20, 21]. Detailed methods are reported elsewhere. Metastatic gastric cancer patients treated at Samsung Medical Centre (Seoul, Korea) with single agent immunotherapy (nivolumab or pembrolizumab) were enrolled in this study. RNAseq was performed on freshly obtained tumor tissues prior to commencing immunotherapy treatment and reported previously [20]. These data along were utilized to associate and analyze *CD8A* and *PDCD1* gene expression with clinical correlates.

#### “3G” gastric cancer cohort analysis

This analysis is a sub-study of a larger dataset of RNAseq of gastric cancer previously reported [22]. Detailed methods are reported elsewhere. Recurrent or metastatic gastric cancer patients treated with platinum fluoropyrimidine doublet chemotherapy in multiple centers in Singapore and South Korea were enrolled in this study. RNAseq was performed on pre-treatment tumor tissue and used for this study.

#### Single-cell RNAseq (scRNAseq) of gastric cancer analysis

##### Sample cohort description

This analysis is a sub-study of a larger dataset of scRNAseq of gastric cancer previously reported [23]. Detailed methods are reported elsewhere. Briefly, patients diagnosed with gastric adenocarcinoma and planned for surgical resection at the National University Hospital, Singapore were enrolled after obtaining written informed consent.

##### Single-cell gene expression quantification and determination of major cell types

Unique molecular identified (UMI) count matrix were first generated for each sample by passing the raw data in the cell ranger software. The count matrix was later used to generate a Seurat object which is used for clustering analysis. UMap was used for two-dimensional representation with ‘RunUMAP’. Differential gene expression for identifying markers of a cluster relative to all other clusters or compared to a specific cluster was determined using the ‘FindAllMarkers’ or ‘FindMarkers’ functions respectively. CD8^+^PD-1^+^ cells were defined as those expressing any level of both *CD8A* and *PDCD1* genes. CD8PD-1_high_ tumors were those with CD8^+^PD-1^+^ proportion higher than the median proportion within the cohort; the rest of the tumors were labelled as CD8PD-1_low_.

### Statistics

Qualitative data in proportions were compared used Fisher’s exact test. Two-sample *t*-test was used to compare parametric quantitative data. Wilcoxon rank-sum test was used to compare non-parametric quantitative data. Spearman’s correlation was used for non-parametric bivariate quantitative comparisons. Kaplan–Meier curves and log-rank test were used for survival analysis. HRs and 95% CIs were evaluated using the Cox proportional hazards regression model. *P*-values of less than 0.05 were considered statistically significant. Statistical analyses were performed using SPSS (IBM Corp., IBM SPSS Statistics for Windows, version 26.0, Armonk, NY, USA) and R V.4.0.5.

## Results

### Clinical samples and baseline characteristics

**Fig. 1:**
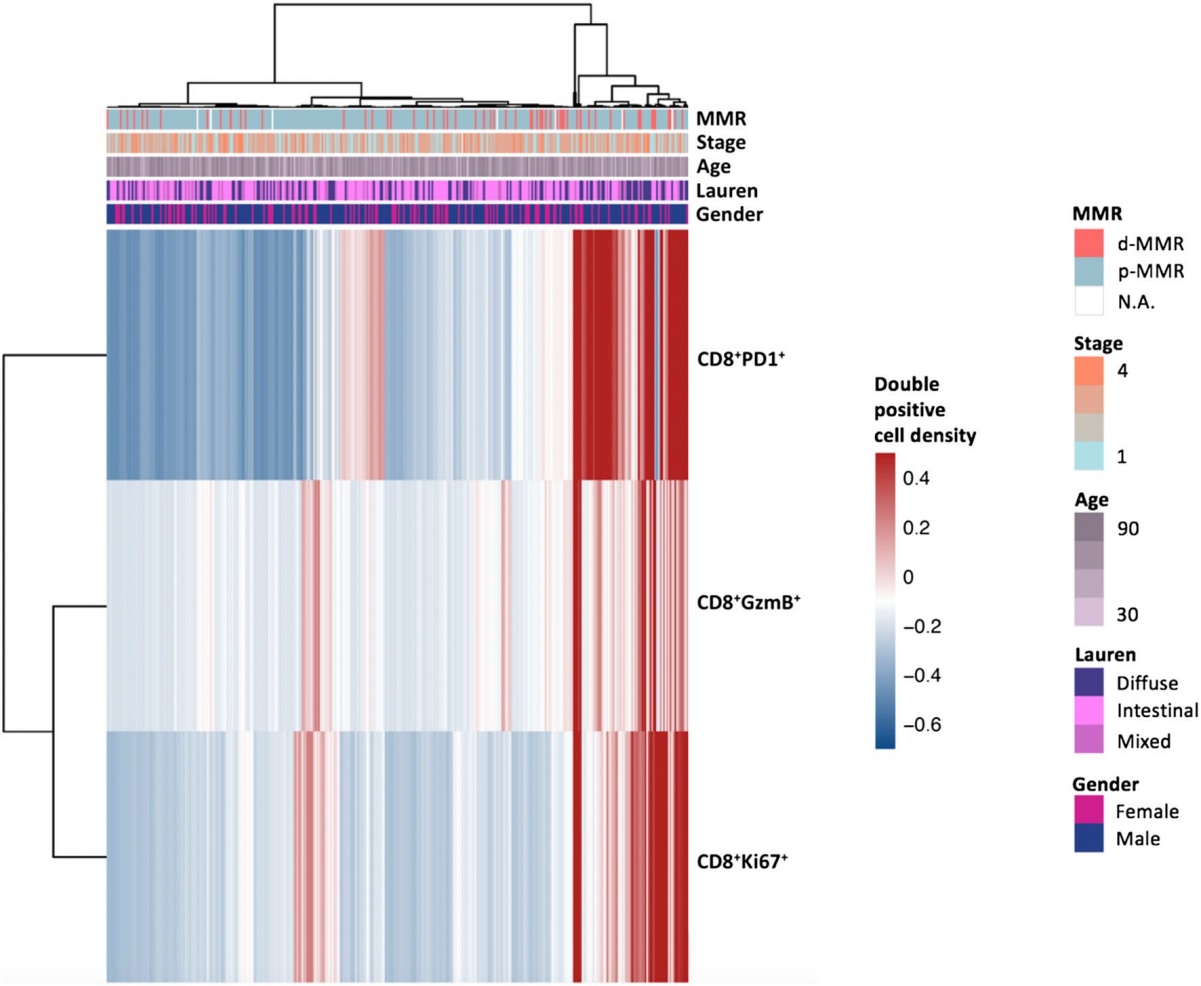
Heatmap of baseline clinicopathological characteristics and association with IHC markers of CD8 T-cell PD-1 expression, activation and proliferation

Tumor samples were obtained from 350 patients with stage I-IV gastric cancer who underwent gastrectomy. Multiplex IHC staining was performed on these samples to evaluate the expression of cytokeratin AE1/AE3, PD-1, CD8, Ki67 and Granzyme B (Supplementary Fig. 2). Samples were also evaluated for MLH1, MSH2, MSH6 and PMS2 protein expression by full slide IHC staining to assess MMR status. Table 1 summarizes the baseline clinicopathological characteristics while Fig. 1 presents their association with IHC markers of CD8 T-cell PD-1 expression, activation and proliferation. Median age at diagnosis was 68 (range 28–93) and 69% were male. A majority of the samples were Stage III and IV (38%, 62% had stage I/II and III/IV respectively). The dataset had a good representation of the different Lauren’s histological subtypes intestinal (56%), diffuse (29%) and mixed (15%) tumors respectively. Deficient mismatch repair protein expression (d-MMR) was found in 16% of samples.

**Table 1:** Baseline clinicopathological characteristics

	All Patients *n* = 350	Percentage, %
Median age, years (range)	68	28–93
Gender		
Male	240	(68.6)
Female	110	(31.4)
Lauren’s		
Intestinal	197	(56.3)
Diffuse	102	(29.1)
Mixed	51	(14.6)
Stage		
I	68	(19.4)
II	66	(18.9)
III	175	(50.0)
IV	40	(11.4)
Differentiation		
Well or moderate	119	(34.0)
Poor	231	(66.0)
MMR status		
p-MMR	286	(81.7)
d-MMR	57	(16.3)
Not available	7	(2.0)

High CD8 T-cell infiltration characterized by high CD8^+^ T-cell to tumor cell ratio was observed in Lauren’s mixed or diffuse type tumors compared to intestinal type tumors (*p* = 0.012), in earlier stage I-II tumors compared to stage III-IV tumors (*p* = 0.036), and in more poorly differentiated tumors (*p* = 0.046) but was not associated with other clinical characteristics. Increased proliferation of tumor infiltrating lymphocytes (TILs), characterized by high proportion of Ki67-positive CD8 (CD8^+^Ki67^+^) cells to tumor cells was more commonly seen in Lauren’s mixed or diffuse type tumors (*p* = 0.002) but was not associated with other clinical characteristics. Increased cytolytic activity characterized by high proportion of Granzyme-B-positive CD8 (CD8^+^GzmB^+^) cells to tumor cells was also more commonly seen in Lauren’s mixed or diffuse type tumors (*p* = 0.005) but again not associated with other clinical characteristics (Supplementary Table 1).

### High CD8 T-cell PD-1 expression was prognostic for overall survival (Fig. 2)

**Fig. 2:**
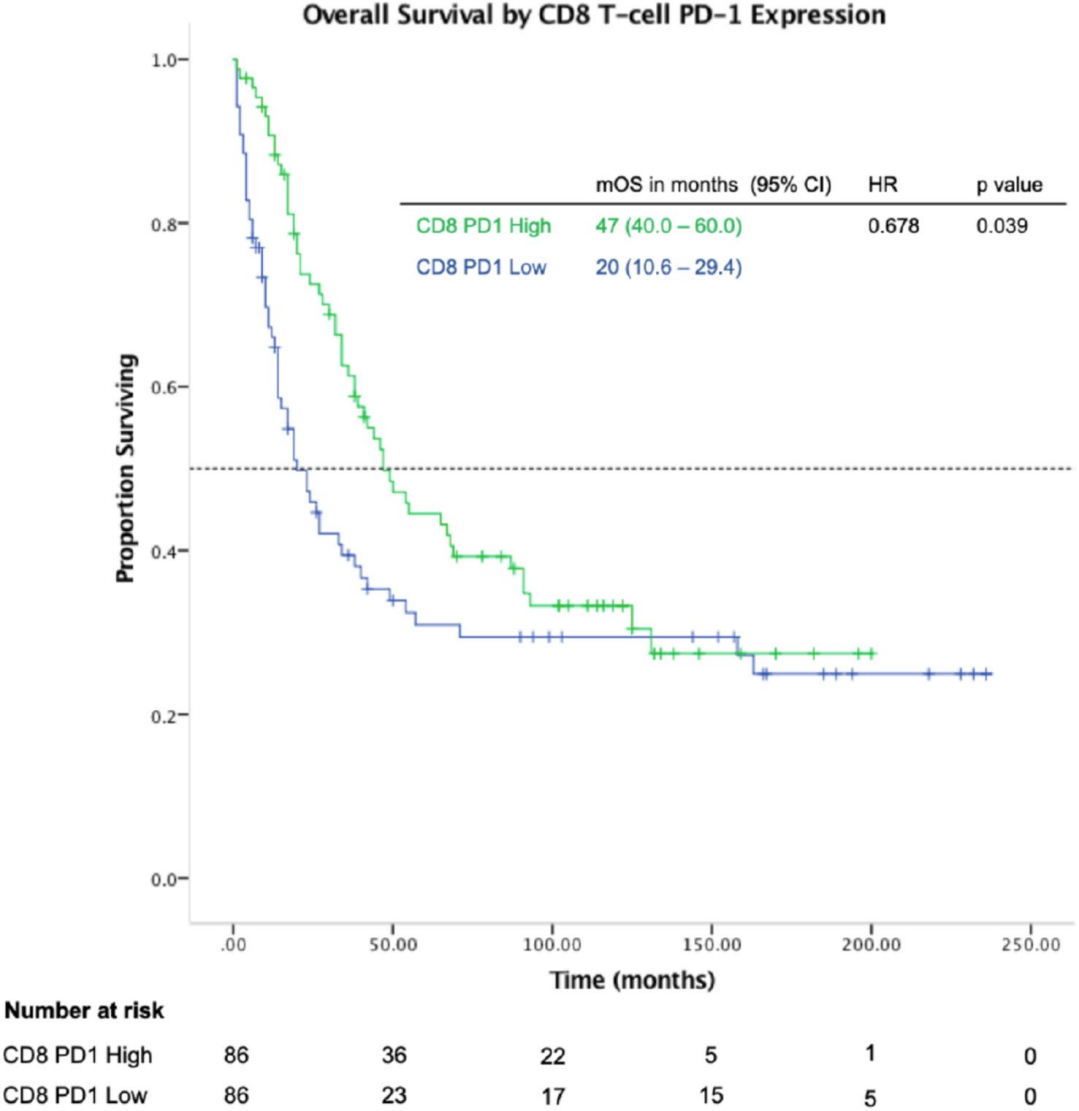
High CD8 T-cell PD-1 expression is associated with improved overall survival in gastric cancer

We studied the effect of increased CD8 T-cell infiltration normalized to tumor cell count and its impact on overall survival and found that there was no significant improvement (HR 0.982; 0.982–1.006, *p* = 0.328). Subsequently, we looked at specific subsets of CD8 T-cells such as PD-1 positive, Ki67 positive and granzyme-B positive CD8 T-cell and found PD-1 positivity to have the greatest effect on prognosis with a robust hazard ratio. Increased proportion of CD8^+^PD-1^+^ T-cells normalized to tumor cell count was prognostic for improved survival with a median OS of 47 vs 20 months (high vs low, HR 0.678; 0.468–0.981; *p* = 0.039; Fig. 2). Proportion of PD-1 positive CD8 T-cells normalized to tumor cell count was highly positively correlated with cytolytic activity (granzyme-B positivity), *r*(347) = 0.714, *p* < 0.001, and activation/proliferation (Ki67 positivity), *r*(347) = 0.798, *p* < 0.001 (Supplementary Table 2).

On univariate analysis, advanced age, advanced tumor stage (III/IV vs I/II), Lauren’s classification (diffuse/mixed vs intestinal), differentiation (poorly vs moderately), were significant prognostic factors for survival. With respect to CD8 T-cell status, high CD8 T-cell PD-1 expression was a significant prognostic factor for survival. High CD8 T-cell cytolytic activity (granzyme-B positivity) and high CD8 T-cell activation (Ki-67 positivity) were also significant prognostic factors for survival. Multivariate analysis using Cox proportional hazards regression demonstrated that high CD8 T-cell PD-1 positivity was an independent prognostic factor. Other independent prognostic factors with significant impact on survival were age, stage and Lauren’s classification (Table 2).

**Table 2:** Univariate and multivariate analysis of clinicopathological factors and impact on OS in gastric cancer patients

Variables	Univariate			Multivariate		
	HR	95% CI	*p* value	HR	95% CI	*p* value
Age (≥ 68 vs < 68)	1.779	1.370–2.361	< 0.001	1.907	1.449–2.510	< 0.001
Stage (III-IV vs I-II)	2.573	1.918–3.453	< 0.001	2.481	1.838–3.350	< 0.001
Lauren (diffuse vs intestinal)	1.534	1.177–2.000	0.002	1.344	1.026–1.760	0.032
Differentiation (poorly vs moderately differentiated)	1.374	1.035–1.824	0.028			
MMR status (deficient vs proficient)	0.709	0.479–1.049	0.086			
Status of CD8 T-cells within tumor						
PD-1 Expression: CD8^+^PD-1^+^/ tumor cell ratio (high vs low)	0.678	0.468–0.981	0.039	0.822	0.680–0.993	0.042
Proliferation: CD8^+^Ki67^+^ / tumor cell ratio (high vs low)	0.783	0.648–0.948	0.012			
Activation: CD8^+^GzmB^+^ / tumor cell ratio (high vs low)	0.741	0.568–0.965	0.026			

### Clinical relevance of CD8 PD-1 positivity in GC treated with chemotherapy and immunotherapy

In the discovery cohort, tumor samples were obtained from chemotherapy naïve patients who underwent gastrectomy in Singapore. Post operatively, patients were offered adjuvant treatment if indicated and underwent surveillance. Of note, at the time of data cut off, none of the patients in our study had received immune checkpoint inhibitors as part of their therapy. We expanded our analysis to other gastric cancer patient cohorts, in particular those treated with various systemic therapies. As multiplex IHC datasets are not available, we analyzed bulk RNAseq data from these studies.

**Fig. 3:**
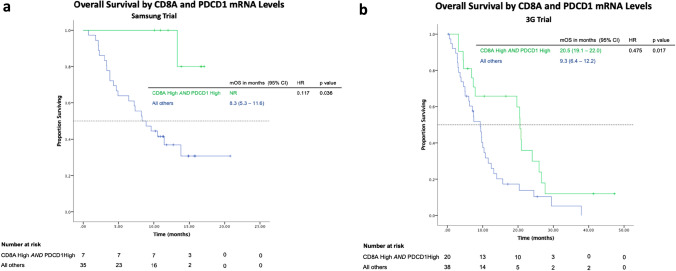
High *CD8A* and High *PDCD1* mRNA levels are associated with improved overall survival in gastric cancer. **a** Overall survival curves of patients enrolled in the Samsung Trial. **b** Overall survival curves of patients enrolled in the “3G” Trial

First, we sought to evaluate the effect of CD8 PD-1 positivity in advanced gastric cancer treated with immune checkpoint inhibitors. We analyzed bulk RNAseq data from gastric cancer patients enrolled in a phase 2 trial conducted at the Samsung Medical Centre, South Korea [21]. Biopsies were obtained from 44 patients who had progressed on at least one cytotoxic regimen prior to treatment with single agent ICIs such as pembrolizumab or nivolumab. Tumors with high *PDCD1* and *CD8A* expression levels were found to have improved overall survival with a median OS of not reached vs 8.3 months (HR 0.117, *p* = 0.036, Fig. 3a). Of note, high *PDCD1* and *CD8A* expression was also associated with improved progression-free survival (PFS), median PFS of 16.1 vs 2.6 months (HR 0.174, *p* = 0.004) and non-progression of disease (non-PD; comprising of patients with complete response [CR], partial response [PR] or stable disease [SD] as best response by RECIST) (*p* = 0.014).

Next we evaluated the effect of CD8 PD-1 positivity in advanced gastric cancer patients treated with chemotherapy. The “3G” trial enrolled patients with locally advanced, metastatic or relapsed gastric cancer treated in Singapore and South Korea with first-line platinum fluoropyrimidine doublet chemotherapy regimen [24]. RNAseq data from 60 tumor samples obtained prior to chemotherapy and also showed improved overall survival in patients with high *PDCD1* and *CD8A* expression, median OS 20.5 vs 9.3 months (HR 0.475, *p* = 0.017, Fig. 3b). Of note, high *PDCD1* and *CD8A* expression was also associated with improved median PFS of 9.4 vs 4.1 months (HR 0.448, *p* = 0.008). However, in terms of tumor responses, high *PDCD1* and *CD8A* expression was not significantly associated with non-progression of disease as best response by RECIST (*p* = 0.458). In patients with high *CD8A* and *PDCD1* expression levels, there were correspondingly high numbers of lymphocytes that stained positive for PD-1 on immunohistochemistry as well (Supplementary Fig. 3).

Lastly, we also evaluated the role of CD8 PD-1 positivity in gastric cancer in non-Asian patients using RNAseq data from the TCGA dataset [25] which sequenced tumors from treatment naïve gastric cancer and showed similar trends to improved overall survival with high *PDCD1* and *CD8A* expression (HR 0.711, *p* = 0.054, Supplementary Fig. 4).

### Interrogation of the tumor microenvironment of tumors driven by CD8 PD-1 positive cells from a single-cell RNAseq gastric cancer dataset

**Fig. 4:**
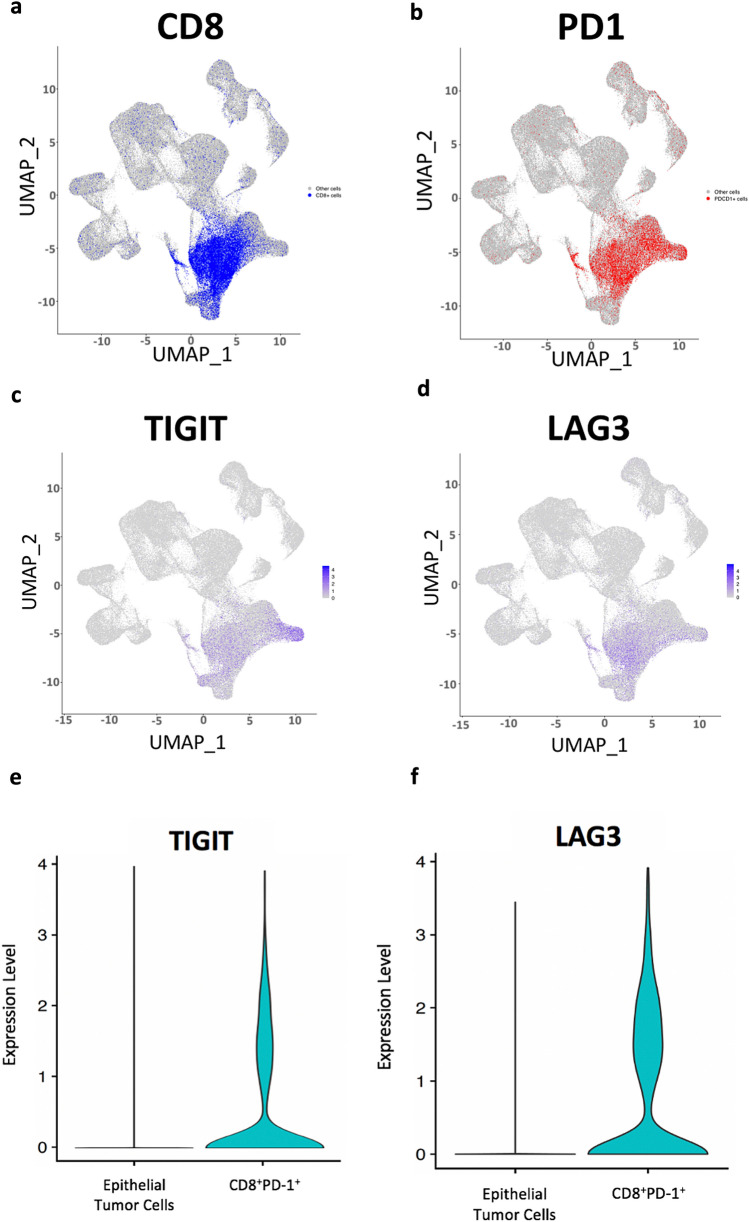
Single-cell RNA sequencing analyses. UMAP plot representation of 152,423 cells from 40 samples delineating cells expressing CD8 **(a)**, PD1 **(b)**, TIGIT **(c)** and LAG3 **(d)**. Violin plots indicating the expression levels of TIGIT **(e)** and LAG3 **(f)**

To have a deeper understanding of the tumor microenvironment compositions of tumors driven by CD8^+^PD-1^+^ cells, we queried the largest dataset of scRNAseq performed in gastric cancer to date [23]. We analyzed 152,423 cells derived from 40 samples (including 11 matched normal samples, and 29 tumor samples). There were no CD8^+^PD-1^+^ cells in the eleven normal samples. We identified 4898 CD8^+^PD-1^+^ cells in tumors, comprising 4.3% of the cellular proportion. More than 90% of CD8^+^PD-1^+^ cells mapped to T-cells and NK-cells as cells of origin. There existed significant variation in inter-sample proportions of CD8^+^PD-1^+^ cells, ranging from 0 to 10.9%, with a of median 5%. Based on the median proportion, we labelled tumors as CD8PD-1_high_ and CD8 + PD-1_low_. We found significant differences in tumor microenvironments between CD8PD-1_high_ and CD8PD-1_low_ tumors (Table 3). For example, T-cell and NK-cell proportions were higher in CD8PD-1_high_ tumors (24% vs 18% and 19% vs 15%, *p* < 0.0001), while macrophage proportions were higher in CD8PD-1_low_ tumors (11% vs 7%, *p* < 0.0001). Further, within the T-cells, the CD8PD-1_high_ tumors had higher proportions of naïve CD8 T-cells (36% vs 26%, *p* < 0.0001) and effector CD8 (9% vs 3%), while CD8PD-1_low_ tumors had higher proportions of naïve CD4 T-cells (26% vs 20%) and helper CD4 T-cells (17% vs 12%). T-reg populations between the two groups were similar (20% vs 21%). We further assessed the CD8^+^PD-1^+^ cells within the scRNAseq dataset for overexpression of PD-1, CTLA-4, TIM-3, LAG-3, 2B4, CD39, CD160, TIGIT, BTLA. CD8^+^PD-1^+^ cells had upregulation of TIGIT and LAG3 (Fig. 4) consistent with features of T-cell repression.

**Table 3:** CD8PD-1_high_ and CD8 + PD-1_low_ tumors have distinct cellular populations and tumor microenvironments

	Median proportions of each cell type (%)											
	T cell	NK cell	Chief Cell	Plasma cell	Fibroblast	B cell	Macro phage	Intestinal metaplasia cell	Pit mucous cell	Endothelial cell	Mast cell	Dendritic cell
Normal tissue	13.5	15.0	14.1	14.1	11.6	1.5	6.1	7.4	6.8	7.5	1.4	0.9
CD8PD-1 high	23.8	18.9	5.7	16.3	8.4	4.0	6.6	5.0	4.2	3.7	2.6	0.9
CD8PD-1 low	18.4	15.2	7.9	12.6	8.1	4.5	10.8	6.4	6.0	5.8	2.8	1.7

## Discussion

Digital multiplex immunohistochemistry/immunofluorescence analysis on the Vectra platform using inForm software was performed to characterize the immune microenvironment of 350 tumor samples from stage I–IV gastric cancer patients who underwent gastrectomy. Using multiplex IHC/immunofluorescence we concurrently evaluated multiple markers which have known predictive and/or prognostic value in numerous solid organ tumors [26–30]. In this study, we demonstrated that a high proportion of PD-1 positive CD8 tumor-infiltrating T-cells was prognostic for overall survival and was associated with a significant 32% reduction in risk of death. CD8 T-cell PD-1 positivity was also highly correlated with CD8 cytolytic activity as well as proliferation/activation. Of note, CD8 T-cell PD-1 positivity was not correlated with other clinicopathological characteristics such as age, stage, Lauren’s type or grade, suggesting that this is possibly an independent prognostic factor in gastric cancer.

Orthogonal analysis of gene expression data from other gastric cancer cohorts including patients who were treated with immunotherapy, first-line platinum doublet chemotherapy further support our findings; at a transcriptional level, combined high *PDCD1* and high *CD8A* gene expression were prognostic for improved overall survival in gastric cancer patients.

We further queried the largest dataset of scRNAseq performed in gastric cancer [23] to evaluate the tumor microenvironment compositions of tumors driven by CD8^+^PD-1^+^ cells. There was significant variation in inter-sample proportions of CD8^+^PD-1^+^ cells, with substantial differences in the tumor microenvironments between CD8PD-1_high_ and CD8PD-1_low_ tumors. CD8PD-1_high_ tumors had higher proportions of both T-cells and NK-cells; the latter have a fundamental role in innate immunity as well as enhancement of anti-tumor adaptive immune responses by secretion of cytokines and retention of immunological memory [31]. CD8PD-1_high_ tumors had higher proportions of naïve CD8 T-cells and effector CD8 T-cells, with lower proportions of macrophages, naïve CD4 T-cells and helper CD4 T-cells.

The role and activation status of infiltrating PD-1 positive CD8 T-cells have been studied in a number of epithelial cancer types such as breast, pancreatic and head and neck tumors showing improved survival outcomes [12–14]. However, the prognostic significance of PD-1 positive CD8 T-cells in gastric cancer is poorly understood, and the available data from studies previously conducted is conflicting with several positive [34, 35] and negative studies [36, 37].

Yu and colleagues recently found that high intratumoral PD-1 positive CD8 T-cells using a cut-off of ≥ 8/HPF was associated with poorer overall survival outcomes as well as poorer efficacy with adjuvant chemotherapy. In this study, the population of CD8 T-cells did not have increased proliferative ability (Ki-67) or cytolytic activity (Granzyme-B), unlike what we observed in our study which may explain some of the differences observed in our study cohort [36]. Saito and colleagues previously reported that higher proportions of PD-1 positive CD8 T-cells present in the circulation portended poorer survival and was also associated with higher stage of disease in gastric cancer [37]. These findings do not directly contradict the findings in our study as they evaluated circulating PD-1 positive CD8 T-cells. Yuan et al. have also previously demonstrated that there is heterogeneity in the proportions of PD-1 positive CD8 T-cells present within the tumor, in the metastatic lymph nodes and in tumor-free lymph nodes from patients with gastric cancer, suggesting that the location of the CD8 T-cells influences functional complexity of this heterogenous cell population.

Shen and colleagues recently evaluated the role of both circulating and tumor-infiltrating PD-1 positive CD8 T-cells; PD-1 positive CD8 T-cells were elevated in gastric tumors compared to normal tissue or circulating peripheral blood mononuclear cells, but elevated percentages of PD-1 positive CD8 cells in gastric tumors were not prognostic for overall survival in their study. Similar to our findings, they observed no significant correlations with clinicopathological features such as stage or LVI/PNI status [38]. Consistent with findings from our study, Park et al. reported that high immune checkpoint receptor expression (including PD-1, LAG3 and TIM3) was mainly expressed in CD8 + T-cells and showed improved survival in stage II and III gastric cancer patients [34]. Another Japanese group [39] evaluated *PD-1* and *CD8* gene expression levels in gastric tumors and found that those with low *PD-1* and *CD8* mRNA had significantly poorer overall survival; these findings support our findings observed in the three orthogonal validation cohorts where high *PDCD1* and *CD8* mRNA levels was associated with improved survival. Apart from these, few other studies have specifically evaluated PD-1 positive CD8 T-cells in gastric cancer.

One limitation of the study was that we did not evaluate the TME architecture in the discovery cohort of patients using spatial techniques as it is not feasible to perform spatial transcriptomics on such a large cohort of samples. Nevertheless, analysis of the orthogonal datasets in particular the scRNAseq findings allowed us to characterize CD8^+^PD-1^+^ T-cells and elucidate differences in the tumor microenvironment between CD8PD-1_high_ and CD8PD-1_low_ tumors. Of interest, we observed high levels of *LAG3* and *TIGIT* in CD8^+^PD-1^+^ cells; these are negative co-stimulatory checkpoints of T-cell activity and mediators of exhaustion. Few studies have specifically evaluated the expression of LAG3 and TIGIT in CD8 T-cells in gastric cancer, however, Park and colleagues previously showed that high immune checkpoint receptor expression (including PD-1, LAG3 and TIM3) in CD8 + T-cells was associated with improved survival in gastric cancer. In d-MMR colorectal cancer, which is the archetypal example of an inflamed TME, there was also deregulated expression of numerous inhibitory immune checkpoint molecules including TIGIT, LAG3 and PD-1 which were more highly expressed in d-MMR compared to p-MMR tumors [32]. Hence, co-expression of T-cell markers of exhaustion in CD8^+^PD-1^+^ cells may paradoxically be associated with improved outcomes and highlight a potential therapeutic vulnerability in this subgroup of tumors with dual-checkpoint inhibition [33].

Our findings on the clinical relevance of CD8 T-cell subset status in gastric tumors are consistent with existing knowledge on the adaptive immune response pathways driven by TILs including the induction of apoptosis in the inflammatory microenvironment of gastric tumors [40]. Spatial and temporal interactions between immune cells and tumor cells appear to be critical for tumor progression [41, 42], with multiple studies reporting that the high concentration of TILs is associated with improved prognosis for gastric cancer [43, 44] and various other tumors [45–47]. The prognostic implications of these activated and highly proliferative CD8 T-cells has only been studied in other tumors like melanoma [30] but not previously studied in gastric cancer.

## Conclusion

This is one of the largest gastric cancer cohorts of multiplex IHC with combined analysis of multiple datasets for orthogonal validation showing that increased PD-1 positive CD8 TILs is associated with improved overall survival. CD8PD-1_high_ tumors have distinct features of an immunologically active, T-cell inflamed tumor immune microenvironment.

## Supplementary Information

Below is the link to the electronic supplementary material.

Supplementary file1 (PDF 46 kb)

Supplementary file2 (PDF 873 kb)

Supplementary file3 (PDF 11538 kb)

Supplementary file4 (PDF 143 kb)

Supplementary file5 (DOCX 20 kb)

Supplementary file6 (DOCX 16 kb)

## Data Availability

All data generated or analyzed during this current study are included in this published article.
