# Supplementary material for: Clinical relevance of PD-1 positive CD8 T-cells in gastric cancer
Source: Gastric Cancer. 2023 Feb 12;26(3):393–404. doi: 10.1007/s10120-023-01364-7 (PMC10115710; doi:10.1007/s10120-023-01364-7)

Supplementary Figure 3: Correspondingly high PD-1 expression by immunohistochemistry in lymphocytes in "3G" cohort samples with high *CD8A* and *PDCD1* expression levels by RNAseq (x400, **a:** H&E, **b:** PD-1)

Supplementary Fig 3a

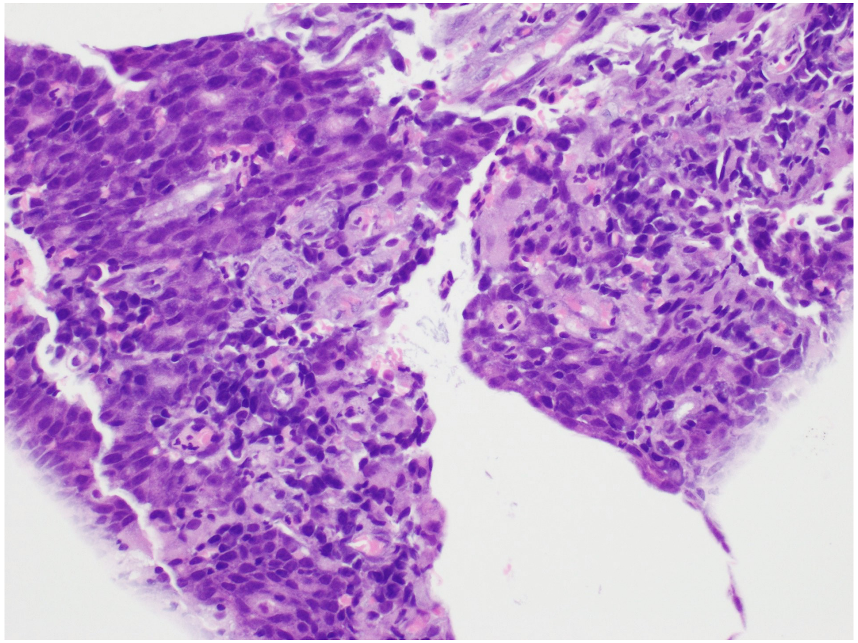

Supplementary Fig 3b

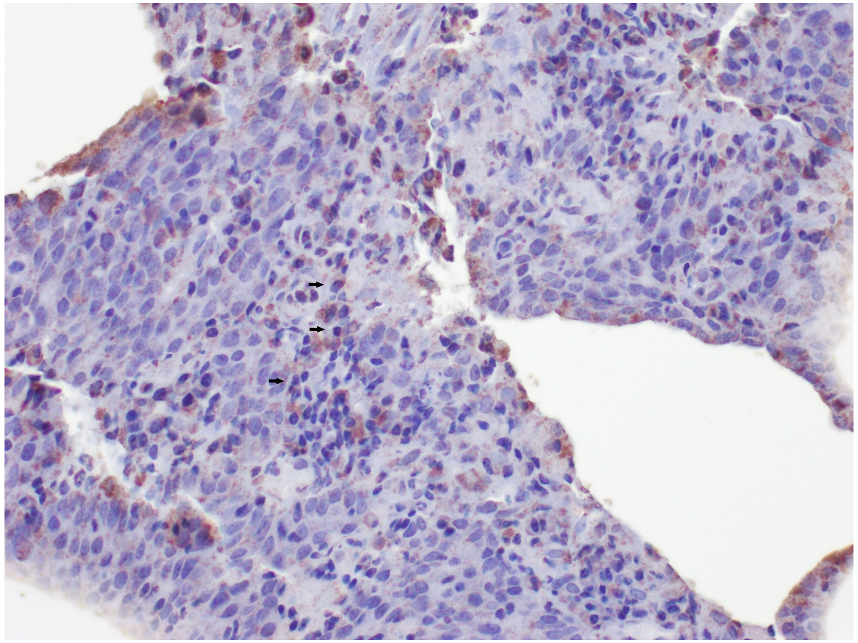

Supplement: Supplementary file 1 — Supplementary file1 (PDF 46 kb) [file 10120_2023_1364_MOESM1_ESM.pdf]
