# Supplementary material for: Clinical relevance of PD-1 positive CD8 T-cells in gastric cancer
Source: Gastric Cancer. 2023 Feb 12;26(3):393–404. doi: 10.1007/s10120-023-01364-7 (PMC10115710; doi:10.1007/s10120-023-01364-7)

Supplementary Figure 2: Correlation and distribution of CD8, PD-1, Granzyme-B, Ki-67 and Cytokeratin in gastric cancer.

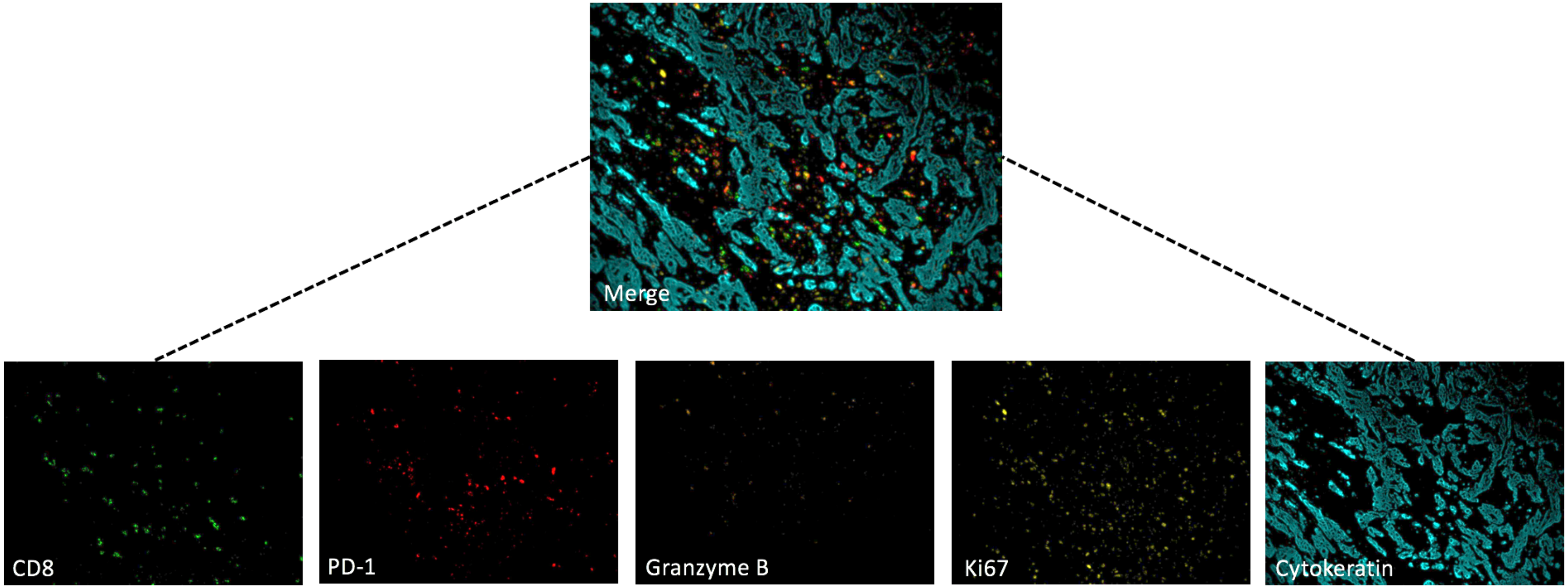

Supplement: Supplementary file 2 — Supplementary file2 (PDF 873 kb) [file 10120_2023_1364_MOESM2_ESM.pdf]
