# Supplementary material for: Clinical relevance of PD-1 positive CD8 T-cells in gastric cancer
Source: Gastric Cancer. 2023 Feb 12;26(3):393–404. doi: 10.1007/s10120-023-01364-7 (PMC10115710; doi:10.1007/s10120-023-01364-7)

Supplementary Figure 1: Principal component analysis biplot of the digital multiplex immunohistochemistry data
Each colour corresponds to each TMA slide (batch). No batch effects were observed based on this analysis.

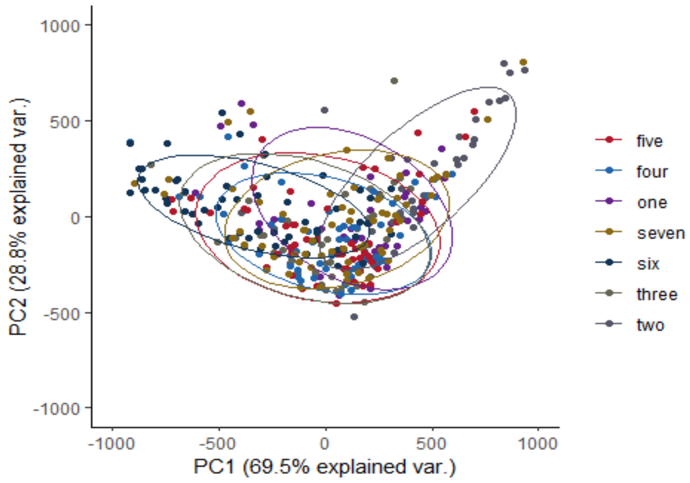

Supplement: Supplementary file 3 — Supplementary file3 (PDF 11538 kb) [file 10120_2023_1364_MOESM3_ESM.pdf]
