# Supplementary material for: Clinical relevance of PD-1 positive CD8 T-cells in gastric cancer
Source: Gastric Cancer. 2023 Feb 12;26(3):393–404. doi: 10.1007/s10120-023-01364-7 (PMC10115710; doi:10.1007/s10120-023-01364-7)
Supplement: Supplementary file 4 — Supplementary file4 (PDF 143 kb) [file 10120_2023_1364_MOESM4_ESM.pdf]

Supplementary Figure 4: High CD8A and PDCD1 mRNA levels has a trend towards improved overall survival in gastric cancer (TCGA's Study of Gastric Adenocarcinoma)

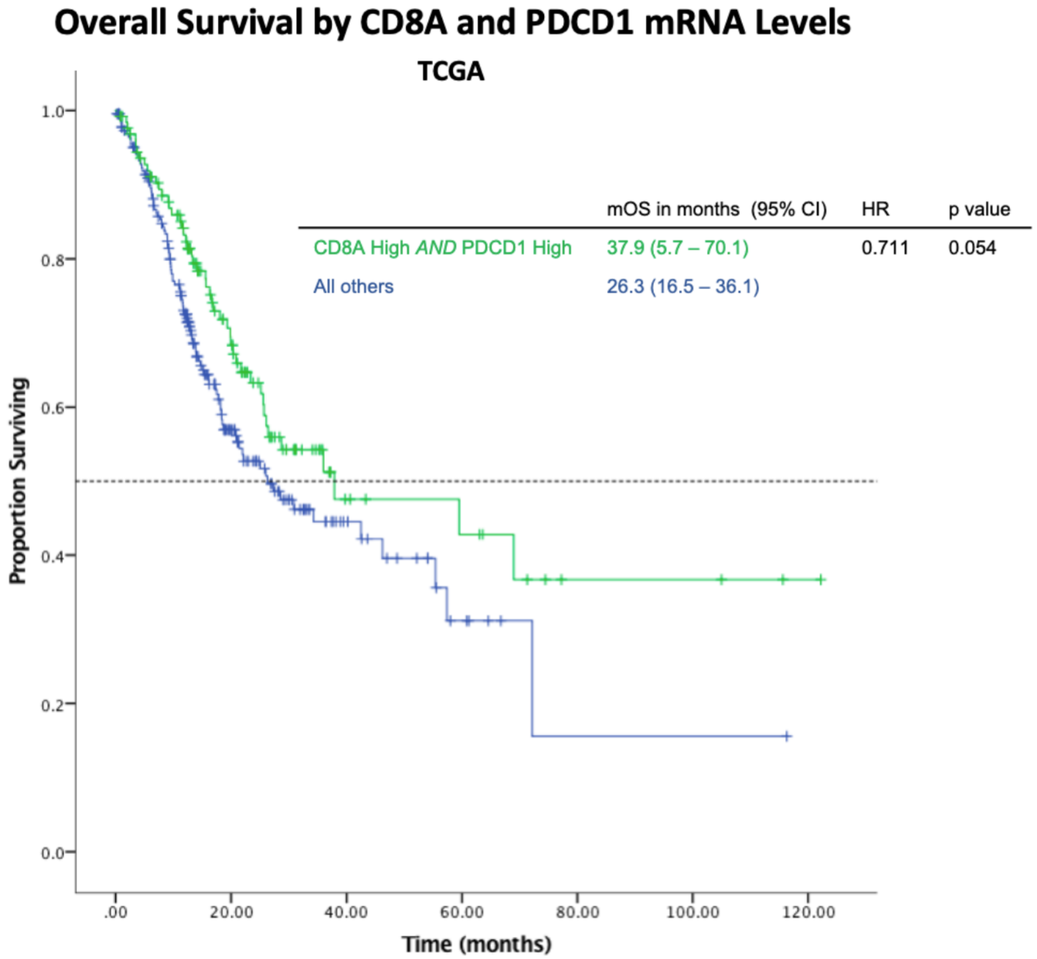

Number at risk

CD8A High *AND* PDCD1High	127	59	12	9	3	3	0
All others	227	73	20	6	1	1	0
