# Supplementary material for: Clinical relevance of PD-1 positive CD8 T-cells in gastric cancer
Source: Gastric Cancer. 2023 Feb 12;26(3):393–404. doi: 10.1007/s10120-023-01364-7 (PMC10115710; doi:10.1007/s10120-023-01364-7)
Supplement: Supplementary file 5 — Supplementary file5 (DOCX 20 kb) [file 10120_2023_1364_MOESM5_ESM.docx]

**Supplementary Table 2: Pearson correlation coefficient between presence of CD8^+^PD-1^+^ T-cells, CD8^+^Ki67^+^ T-cells and CD8^+^GzmB^+^ T-cells.**

|  | CD8^+^PD-1^+^ | CD8^+^Ki67^+^ | CD8^+^GzmB^+^ | |
| --- | --- | --- | --- | --- |
| CD8^+^PD-1^+^ | 1 |  |  |  |
| CD8^+^Ki67^+^ | r= 0.798  p<0.001 | 1 |  |  |
| CD8^+^GzmB^+^ | r= 0.714  p<0.001 | r= 0.916  p<0.001 | 1 |  |
