# Supplementary material for: Clinical relevance of PD-1 positive CD8 T-cells in gastric cancer
Source: Gastric Cancer. 2023 Feb 12;26(3):393–404. doi: 10.1007/s10120-023-01364-7 (PMC10115710; doi:10.1007/s10120-023-01364-7)
Supplement: Supplementary file 6 — Supplementary file6 (DOCX 16 kb) [file 10120_2023_1364_MOESM6_ESM.docx]

**Supplementary Table 1: Baseline clinicopathological characteristics and association with CD8 T cell infiltration and IHC markers of CD8 T-cell proliferation and cytolytic activity.**

| **All Patients**  **n=355, (%)** | | **Number of Pts (% )** | | | | | | | | | | | | |
| --- | --- | --- | --- | --- | --- | --- | --- | --- | --- | --- | --- | --- | --- | --- |
|  |  | **CD8 T-cell infiltration** | | | | **CD8 T-cell proliferation (Ki-67 expression)** | | | | **CD8 T-cell cytolytic activity (Granzyme-B)** | | | | |
|  |  | **Low** | **Int** | **High** | **p** | **Low** | **Int** | **High** | **p** | **Low** | **Int** | **High** | **p** |  |
| Median age, years (range) | 68 (28-93) | 71 (37-93) | 69 (28-90) | 65 (30-93) | 0.053 | 69 (37-93) | 70 (29-89) | 66 (28-93) | 0.286 | 71 (37-93) | 69 (29-89) | 68 (28-93) | 0.788 |  |
| Gender  Male  Female | 240 (68.6)  110 (31.4) | 84 (35.0)  41 (37.3) | 99 (41.3)  53 (48.2) | 57 (23.8)  16 (14.5) | 0.135 | 53 (22.1)  34 (30.9) | 124 (51.7)  52 (47.3) | 63 (26.3)  24 (21.8) | 0.197 | 55 (22.9)  32 (29.1) | 124 (51.7)  52 (47.3) | 61 (25.4)  26 (23.6) | 0.462 |  |
| Lauren’s  Intestinal  Diffuse/Mixed | 197 (56.3)  102 (29.1) / 51 (14.6) | 83 (42.1)  42 (27.5) | 80 (40.6)  72 (47.1) | 34 (17.3)  39 (25.5) | **0.012** | 58 (29.4)  29 (19.0) | 103 (52.3)  73 (47.7) | 36 (18.3)  51 (33.3) | **0.002** | 52 (26.4)  35 (22.9) | 109 (55.3)  67 (43.8) | 36 (18.3)  51 (33.3) | **0.005** |  |
| Stage  I / II  III /IV | 68(19.4) / 66 (18.9)  175 (50.0) / 40 (11.4) | 47 (35.1)  78 (36.1) | 50 (37.3)  102 (47.2) | 37 (27.6)  36 (16.7) | **0.036** | 33 (24.6)  54 (25.0) | 62 (46.3)  114 (52.8) | 39 (29.1)  48 (22.2) | 0.320 | 37 (27.6)  50 (23.1) | 66 (49.3)  110 (50.9) | 31 (23.1)  56 (25.9) | 0.616 |  |
| Differentiation  Well or mod  Poor | 119 (34.0)  231 (66.0) | 53 (44.5)  72 (31.2) | 44 (37.0)  108 (46.8) | 22 (18.5)  51 (22.1) | **0.046** | 33 (27.7)  54 (23.4) | 63 (52.9)  113 (48.9) | 23 (19.3)  64 (27.7) | 0.215 | 34 (28.6)  53 (22.9) | 64 (53.8)  112 (48.5) | 21 (17.6)  66 (28.6) | 0.074 |  |
| MMR status  p-MMR  d-MMR  Nil | 286 (81.7)  57 (16.3)  7 (2.0) | 102 (35.7)  22 (38.6) | 129 (45.1)  19 (33.3) | 55 (19.2)  16 (28.1) | 0.180 | 71 (24.8)  16 (28.1) | 143 (50.0)  28 (49.1) | 72 (25.2)  13 (22.8) | 0.855 | 73 (25.5)  14 (24.6) | 148 (51.7)  24 (42.1) | 65 (22.7)  19 (33.3) | 0.214 |  |
